# Single-Bout Strength: Acute Mental Health Responses to Resistance Training in Active Adults

**DOI:** 10.3390/sports13070221

**Published:** 2025-07-07

**Authors:** Manuel Amore, Adolfo Alfarano, Vincenzo Sorgente, Giulia Panconi, Sara Guarducci, Riccardo Bravi, Diego Minciacchi

**Affiliations:** 1 Kinesiology and Motor Control (Ki.Mo.Co.) Laboratory, Department of Experimental and Clinical Medicine, Physiological Sciences Section, University of Florence, 50134 Florence, Italy; adolfo.alfarano@edu.unifi.it (A.A.); vincenzo.sorgente@unifi.it (V.S.); giulia.panconi@unifi.it (G.P.); riccardo.bravi@unifi.it (R.B.); 2 Department of Information Engineering, University of Florence, 50121 Florence, Italy; sara.guarducci@unifi.it

**Keywords:** resistance training, anxiety, depression, acute exercise, mental health, athletes, HADS, non-pharmacological intervention

## Abstract

Background: Emerging evidence highlights the role of physical exercise as a non-pharmacological intervention for reducing symptoms of anxiety and depression. While most research has focused on aerobic modalities and chronic training programs, the acute psychological impact of resistance training (RT)—particularly in healthy, active individuals—remains underexplored. This study addresses this gap by evaluating the immediate effects of a single RT session on anxiety and depression in healthy, active individuals. Methods: Fifty-six healthy, physically active participants (43 males, 13 females; M age = 24.41 ± 4.41 years) were randomly assigned to an experimental group (RT, *n* = 30) or a control group (stretching/mobility, *n* = 26). The RT session included multi-joint exercises performed at 70–75% 1RM, while the control session consisted of non-load-based mobility and flexibility exercises. Psychological responses were measured immediately before and five minutes after the session using the Hospital Anxiety and Depression Scale (HADS), which includes subscales for anxiety (HADS-A) and depression (HADS-D). Results: Non-parametric within-group analysis (Wilcoxon Signed-Rank Test) revealed a significant reduction in anxiety scores in the RT group (Z = −3.3, *p* < 0.001, r = −0.7), and a moderate but significant decrease in depression (Z = −2.8, *p* = 0.005, r = −0.6). No significant changes were observed in the control group for either variable. Between-group comparisons (Mann–Whitney U) showed significantly greater reductions in anxiety in the RT group (*p* = 0.021), while differences in depression deltas were not significant. A Quade ANCOVA confirmed that group assignment is significantly predictive for post-intervention anxiety levels (F(1, 54) = 8.46, *p* = 0.005), controlling for baseline values. Conclusions: A single session of moderate-to-high-intensity resistance training can acutely reduce anxiety symptoms in healthy physically active individuals. The effect on depressive symptoms appears more modest and variable, suggesting differential sensitivity to acute exercise stimuli. These findings support the use of RT not only for long-term psychological health but also as a feasible short-term intervention for emotional regulation in healthy active populations.

## 1. Introduction

The benefits of physical activity on mental health are increasingly recognized across a wide range of sport settings, from recreational participation to elite competition [1]. Interest has grown in physical exercise as a complementary or even alternative approach to pharmacological and psychotherapeutic treatments for anxiety and depression [2,3,4]. This interest is particularly relevant for healthy physically active individuals, who often engage in regular training routines and may be exposed to various lifestyle stressors—such as time constraints, occupational demands, or personal expectations—that can impact psychological well-being. These stressors may contribute to fluctuations in mood and affective state, potentially interfering with motivation, concentration, and overall adherence to training or healthy routines [5,6].

Empirical evidence has consistently shown that physical exercise can reduce symptoms of anxiety and depression. Aerobic activities such as running, swimming, cycling, and brisk walking—as well as mind–body practices like yoga—have been associated with significant improvements in mood and reductions in state anxiety, even following a single session. These acute effects have been observed in both clinical and non-clinical populations and may last for several hours post-exercise [3,7,8].

In contrast, lower-intensity and low-impact activities such as stretching, Tai Chi, or Pilates have shown more modest benefits, primarily in terms of relaxation and perceived well-being, particularly when practiced regularly in meditative or therapeutic contexts [9,10]. However, these modalities typically fail to elicit sufficient neuromuscular and metabolic stimulation to activate the neurobiological mechanisms most closely associated with mood regulation [11]. In this regard, exercise intensity emerges as a critical variable. More demanding forms of training appear to exert greater psychological effects by generating more robust physiological stress and subsequent adaptive responses [12].

Among higher-intensity, anaerobic modalities, resistance training (RT) has received growing attention for its potential to reduce affective symptoms, even after a single session [13,14,15]. Unlike aerobic activity, RT—particularly when performed at moderate-to-high intensity—imposes substantial neuromuscular and metabolic demands, which may lead to distinct psychophysiological effects [16,17,18,19,20].

Physiologically, the acute mental health benefits of RT are thought to be mediated by a range of neuroendocrine, neurochemical, and immune mechanisms. These include modulation of the hypothalamic–pituitary–adrenal (HPA) axis and associated cortisol responses, which are typically more pronounced following RT than aerobic activities of similar duration [21]. RT also induces the release of mood-related monoamines such as serotonin, dopamine, and noradrenaline [22], alongside elevations in brain-derived neurotrophic factor (BDNF), which promotes neuroplasticity and mood stabilization [11,23]. Additionally, RT stimulates the release of β-endorphins and contributes to an anti-inflammatory environment by reducing pro-inflammatory cytokines (e.g., IL-6, TNF-α) and enhancing anti-inflammatory markers such as IL-10 [15]. While aerobic exercise can also elicit some of these responses, the intensity-dependent nature of RT may result in greater or more rapid activation of these biological pathways [24]. This may explain the stronger anxiolytic and mood-stabilizing effects observed in certain RT protocols when compared with aerobic modalities.

Despite this growing body of evidence, several important gaps remain. First, most studies to date have focused on chronic exercise interventions, largely overlooking the potential acute benefits of a single RT session [24,25,26,27]. Second, the majority of research has been conducted on sedentary or clinical populations, leaving healthy physically active individuals underrepresented [13,15,25,26,28]. This is particularly surprising given the known association between lifestyle stressors and psychological distress, even in non-clinical populations. Moreover, existing studies often suffer from methodological limitations such as lack of ecological validity, inadequate control conditions, or failure to consider contextual factors such as time of day, recent workload, or emotional baseline.

Based on existing literature and the outlined physiological mechanisms, we formulated the following hypotheses: (1) participants undergoing a single session of moderate-to-high-intensity resistance training (RT) would exhibit a significant reduction in anxiety and depression symptoms immediately post-intervention; (2) no significant changes would occur in the control group performing stretching and mobility exercises, given their lower neuromuscular and metabolic demand; (3) the magnitude of psychological improvement—particularly regarding anxiety—would be greater in the RT group compared to the control group.

## 2. Materials and Methods

### 2.1. Participants

A total of 56 participants (comprising 43 males and 13 females) with a mean age of 24.41 years (SD = 4.41) were recruited for this study. An a priori power analysis was conducted using G*Power (version 3.1.9.6) to determine the required sample size. Based on an expected large effect size (Cohen’s d = 0.70), an alpha level of 0.05, and a desired power of 0.80, the analysis indicated that a minimum of N = 26 participants per group would be needed for a one-tailed independent samples *t*-test; therefore, we conservatively recruited a total of 56 competitive athletes to participate in this study. The participants were divided into two groups: 30 were allocated to the experimental (E) group and 26 were allocated to the control (C) group. All participants were healthy and physically active individuals regularly engaged in sports practice, with a minimum of two training sessions per week, including those in the C group. Participants did not belong to a specific subpopulation (e.g., athletes of a particular discipline or academic cohort), but were selected based on general criteria of being healthy and physically active. Participants were selected using a purposive sampling method, targeting healthy and physically active individuals engaged in regular sports practice. No familiarization session was required prior to the intervention, as all participants were already familiar with the exercises involved. Moreover, individual one-repetition maximums (1RMs) had been previously assessed, and all training sessions were conducted under the supervision of qualified experts. Written informed consent was obtained from all participants prior to the start of the study. Subjects were randomly assigned using the Random Allocation Software (https://random-allocation-software.software.informer.com, accessed on 26 May 2025, version number 1.0) to one of two groups: a resistance training, the E group, and a C group performing non-load-based physical activity. The study protocol was approved by the Institutional Ethics Committee (Area Vasta Centro AOU Careggi, Florence, Italy—ref:17768_oss). Before the start of the experiments, participants provided written informed consent.

### 2.2. Experimental Procedure

The resistance training session was standardized across participants and began with a general warm-up (10–15 min of low-intensity treadmill walking or jogging), followed by a specific warm-up, consisting of a series of progressively heavier machine-based and free-weight exercises. The average rest between exercises was approximately 2 min and 30 s.

**Figure 1:**
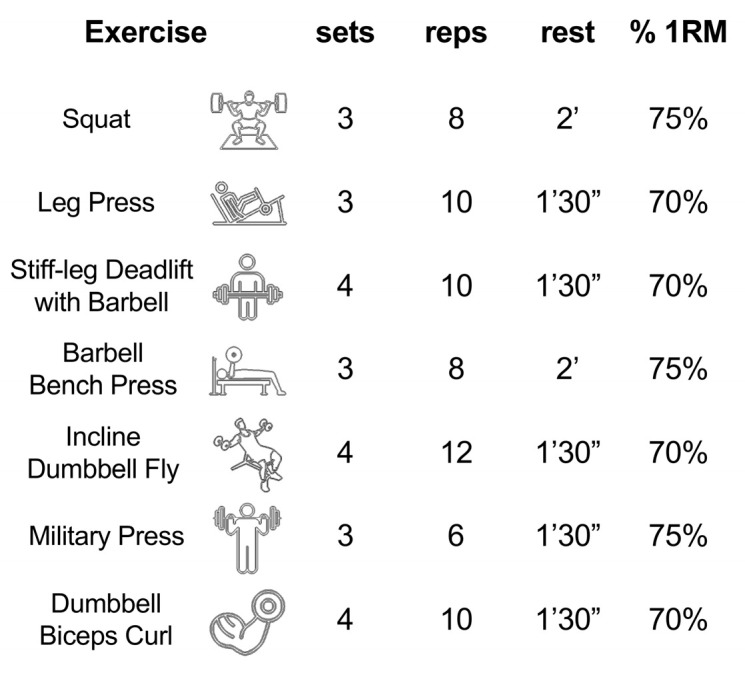
Resistance training protocol implemented by the experimental group: exercises were performed at 70% and 75% of 1RM of each participant.

Figure 1 illustrates the resistance training program followed by the experimental group.

The experimental RT session was structured according to established recommendations for acute resistance training protocols capable of eliciting neuromuscular, endocrine, and affective responses. The use of multi-joint exercises performed at 70–75% of 1RM, with 3–4 sets and moderate rest intervals (90–120 s), aligns with evidence-based guidelines used in previous studies on acute psychological and physiological adaptations [21,29,30].

### 2.3. Group C

Participants in the control group were instructed to adhere to a stretching and mobility protocol. The duration of the session was 60 min and did not involve the use of external resistance. The stretching and mobility protocol was standardized across participants and was preceded by a general warm-up (10–15 min of low-intensity treadmill walking or jogging). The training session was divided into three phases, beginning with dynamic activation, followed by joint mobility and concluding with static stretching.

### 2.4. Psychological Assessment

Participants completed the Hospital Anxiety and Depression Scale (HADS) [31,32] both immediately before and five minutes after their assigned activity. The HADS is a 14-item self-report instrument that assesses anxiety and depression on two subscales (HADS-A and HADS-D), each consisting of 7 items rated on a 4-point Likert scale (0–3). Higher scores indicate more severe symptoms. Previous studies reported good internal consistency for HADS, with Cronbach’s α ranging from 0.80 to 0.93 for HADS-A and from 0.76 to 0.90 for HADS-D in non-clinical samples [33]. The Hospital Anxiety and Depression Scale (HADS) has robust psychometric properties and is frequently used in both clinical and non-clinical populations, including physically active individuals [31,33]. The scale is designed to minimize the influence of somatic symptoms—such as fatigue or muscle soreness—that could confound results in exercise studies [34]. Its sensitivity to short-term emotional changes makes it appropriate for detecting acute variations in anxiety and depression following a single exercise session [35].

### 2.5. Statistical Analysis

All analyses were performed using IBM SPSS Statistics, version 30.0. Non-parametric methods were employed due to violations of normality (confirmed via Shapiro–Wilk tests, *p* < 0.05). Within-group changes from pre- to post-intervention were assessed using the Wilcoxon Signed-Rank Test. Between-group comparisons of score changes (Δ = post − pre) were conducted using the Mann–Whitney U Test. In addition, the Mann–Whitney U test was used to explore between-group differences, such as potential sex effects. Due to violations of normality assumptions and the need to control for baseline values, the Quade non-parametric analysis of covariance (Quade’s ANCOVA) was conducted for both anxiety and depression scores. This method ranks both the covariate (pre-intervention scores) and the outcome (post-intervention scores), removes the linear effect of the covariate, and compares the adjusted residuals between groups using an ANOVA framework. The Quade’s ANCOVA approach is considered appropriate for small samples and non-normally distributed data [36]. Statistical significance was set at α = 0.05, and effect sizes were interpreted according to Cohen’s criteria: r = 0.10 as small, r = 0.30 as medium, and r = 0.50 or above as large effects.

## 3. Results

Prior to conducting the core analyses, the normality of the distributions of anxiety and depression scores (both pre- and post-intervention) within the two groups was examined. The results of the Shapiro–Wilk test demonstrated in all conditions a significant deviation from normality (*p* < 0.05), thus providing a rationale for the utilization of non-parametric tests in data analysis. At baseline, males and females did not differ significantly on any of the self-reported psychological measures (all *p* > 0.05).

The analysis of intra-group variation for HADS-A demonstrated significant effects within the E group. The Wilcoxon Signed-Rank Test revealed a substantial decrease in anxiety levels following a single-bout of RT (median pre = 6, median post = 4), with a Z value of −3.3 (*p* = 0.001) and a large effect size (r = −0.7). Conversely, within the C group which comprised participants engaging in physical activity without external loads, the decline in anxiety scores did not attain statistical significance (median pre = 6, median post = 5), Z = −1.5, *p* = 0.139, with a medium effect size (r = −0.4).

A similar trend was also identified in HADS-D scores. In the E group a significant reduction in post-intervention scores was observed (median pre = 4.5, median post = 3.5), with a Z score of −2.8, a *p* value of 0.005, and a large effect size (r = −0.6). Conversely, within the C group, the observed enhancement did not attain statistical significance (median pre = 5.5, median post = 4), Z = −1.5, *p* = 0.135, with a medium effect size (r = −0.4). Data are reported in table format (Table 1) to ease reading.

To further facilitate a comparison of the variations between the groups, the differences (deltas) between the post and pre scores for each subject were calculated. The application of the Mann–Whitney test to the comparison of anxiety deltas revealed a significant difference between the groups (U = 254, Z = −2.31, *p* = 0.021), indicating a medium effect size (r = 0.31). In contrast, the analysis revealed no statistically significant difference in the delta depression scores between the two groups (U = 325.5, Z = −1.07, *p* = 0.283), with a small effect size (r = 0.14). This latter finding indicates that the groups did not differ significantly in terms of depressive symptoms.

We also conducted a Quade’s ANCOVA that revealed a statistically significant effect of group on post-intervention anxiety scores, F(1, 54) = 8.46, *p* = 0.005, indicating that participants in the E group exhibited greater reductions in anxiety compared to those in the C group, after adjusting for baseline anxiety levels. In contrast, the same analysis conducted on depression scores did not show a significant between-group effect, F(1, 54) = 1.47, *p* = 0.230, suggesting that the acute resistance training session did not lead to a measurable short-term improvement in depressive symptoms relative to the control condition.

**Figure 2:**
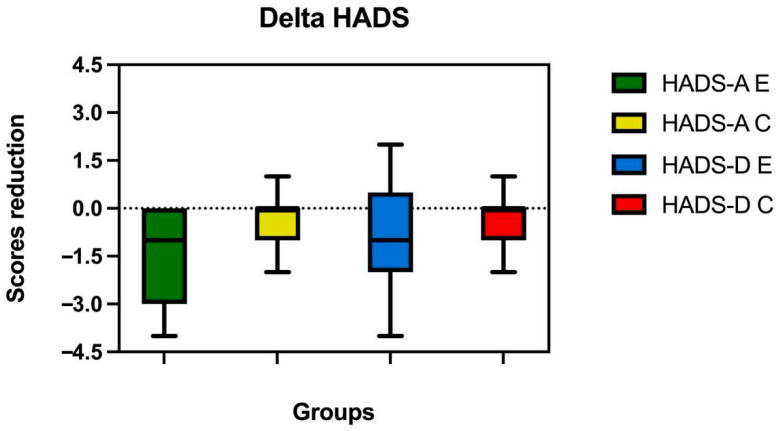
This figure presents boxplots showing the distribution of change (Δ = post − pre) in HADS-A and HADS-D scores for anxiety and depression in both the E and C groups. Negative values represent a reduction in symptom severity. The boxes indicate the interquartile range (IQR), the horizontal line within each box represents the median, and the whiskers extend to the most extreme data points within 1.5× IQR from the lower and upper quartiles (Tukey’s method).

Figure 2 illustrates changes (Δ = post − pre) in depression and anxiety scores for both groups.

## 4. Discussion

This study examined the acute psychological effects of a single session of RT on symptoms of anxiety and depression in healthy physically active individuals. We observed a significant immediate reduction of anxiety symptoms in the experimental RT group compared to the control group engaged in non-resistance physical activity. These findings contribute to the growing body of literature suggesting that even brief bouts of RT may produce measurable psychological benefits [14].

Testing for sex differences is a common and relevant step in psychological and exercise-related research, given the well-documented biological and psychosocial distinctions between men and women in stress reactivity, hormonal profiles (e.g., cortisol, estrogen), and vulnerability to anxiety and depressive symptoms [37,38]. These differences may influence both baseline affective states and responses to physical exercise interventions. However, the absence of significant sex-based differences in our sample suggests a comparable psychological status across genders at the start of the intervention. Accordingly, and following the approach of previous studies that have collapsed groups in the absence of such differences [2,25], subsequent analyses were conducted on the pooled sample regardless of sex, allowing for a more parsimonious interpretation and increased statistical power.

Our results are consistent with previous research indicating that acute bouts of physical activity can induce positive mood changes and reduce anxiety symptoms. For instance, a systematic review found that a single session of RT can lead to immediate reductions in state anxiety [3], with effects lasting up to 2 h post-exercise, especially in individuals with elevated baseline symptoms. Similarly, other studies [29,39] reported significant improvements in mood following a single bout of moderate-intensity RT, with reductions in tension, anger, and confusion.

On the other hand, participants in the C group, who engaged in non-resistance, low-intensity physical activities such as stretching, mobility, and Pilates, did not show significant reductions in anxiety or depression scores, in contrast to what previous research has shown [3,9,10]. However, it is important to note that such benefits are often contingent upon the frequency and duration of the intervention. Indeed, low-impact activities may require sustained and regular practice over longer periods to elicit meaningful changes in mood or anxiety regulation [2]. This temporal aspect may explain the absence of significant effects found in the present study, which employed a short intervention window. This finding highlights an important distinction: not all forms of physical activity yield equivalent acute psychological effects. While low-intensity activities such as stretching or mobility work may support general well-being, they often lack the neuromuscular and metabolic demands necessary to activate the neurobiological mechanisms responsible for acute affect regulation [2,11].

The differential effects observed between groups may be explained by psychophysiological mechanisms that are acutely responsive to exercise intensity and complexity. Moderate-to-high-intensity RT, as applied in our protocol, is known to activate several pathways involved in emotional regulation. These include modulation of the HPA axis and cortisol levels [16,17,21], increased availability of mood-related monoamines such as serotonin, dopamine, and norepinephrine [22,40], and BDNF [11,23,41].

Additionally, RT may exert acute anti-inflammatory effects by reducing pro-inflammatory cytokines and enhancing anti-inflammatory responses [42], thus contributing to a more favorable biochemical setting for psychological well-being. Recent perspectives also point to the involvement of IGF-1 signaling, cerebrovascular adaptations, and respiratory control during resistance exercise as possible contributors to these immediate affective benefits [15]. Altogether the convergence of neurochemical, endocrine, and immune processes provides a plausible explanation for the observed reduction in anxiety scores in our RT group.

However, while the effect on anxiety was robust, the reduction in depressive symptoms was smaller and less consistent across analyses. This asymmetry is not unexpected: previous works [7,43] have shown that depression tends to be less reactive to acute exercise and may require repeated sessions or longer interventions to produce significant shifts. This difference may reflect distinct neurobiological timeframes for anxiety and depression responses, with anxiety being more sensitive to transient physiological perturbations and depression requiring more sustained neuroplastic and psychosocial engagement [13].

It is also important to consider the role of training familiarity and environmental factors in shaping the affective responses observed [44]. All participants had prior experience with RT, which likely enhanced their sense of competence and self-efficacy during the session [45,46]. The training took place in a familiar and non-evaluative setting, under supervision, with clearly structured tasks and appropriate rest intervals. These elements might have mitigated perceived stress, allowing even a demanding protocol to be experienced as controllable and positively engaging rather than aversive [47]. The perception of mastery, rather than threat or failure, has been shown to moderate the emotional effects of physical exertion [12], and likely contributed to the favorable outcomes we observed.

These contextual considerations assume particular relevance when interpreting our results in relation to literature, which suggests greater psychological benefits from low-to-moderate-intensity RT (e.g., 40–60% 1RM), especially when combined with longer rest intervals [13]. While such protocols may indeed reduce anxiety under certain conditions, our findings indicate that more intense training—when performed under the right contextual circumstances—can also yield positive effects. The key, therefore, may not lie in intensity alone, but in how intensity is managed, perceived, and supported by the training environment and the individual’s familiarity with the task [48,49].

Altogether, our findings support a more complex and individualized model of affective response to RT. Rather than advocating a single “optimal” intensity, the data suggest that several factors—including physiological load, perceived exertion, contextual safety, and prior experience—interact dynamically to shape the psychological impact of a workout. This model helps reconcile our results with previous studies and highlights the potential of RT as an accessible, immediate, and multifactorial effective strategy for emotional regulation, even when delivered as a single session [12].

Nonetheless, some limitations should be acknowledged. First, the sample included only physically active individuals, which limits generalizability. Sedentary individuals benefit even more from RT, while elite athletes, due to their high stress and performance demands, respond differently showing even greater psychological gains. Second, the exclusive reliance on self-report measures though being more ecologically compatible introduces potential biases. Future studies could incorporate physiological markers such as salivary cortisol, BDNF, or heart rate variability to provide a comprehensive understanding of underlying mechanisms. Third, several contextual variables—such as sleep quality and duration, recent nutritional intake, time of day, and acute emotional state—may have influenced participants’ affective responses. While participants received standardized pre-session instructions (e.g., to avoid intense physical activity, maintain hydration, and refrain from caffeine), we did not implement formal monitoring or systematic recording of these factors. Given the growing evidence that such variables can modulate acute mood responses to exercise, their uncontrolled variation represents a potential confounding influence. Future research should incorporate objective or semi-structured assessments—such as sleep and diet logs, mood scales, and time-of-day standardization—to account for these effects more rigorously. This would improve internal validity and help isolate the specific contribution of exercise type and intensity to observed psychological outcomes.

Ultimately, this study provides new evidence that a single session of structured, moderately intense RT can acutely reduce anxiety levels and depression in healthy, physically active individuals. The findings highlight the relevance of exercise intensity and individual–contextual interaction in eliciting psychological benefits, suggesting that RT may serve as a practical short-term strategy for affect regulation, even outside of clinical or elite athletic populations. Pre-performance routines (PPRs), such as imagery, self-talk, and controlled breathing, help athletes manage arousal and anxiety before competition. Grounded in the IZOF model [50], these routines assist athletes in reaching their optimal psychological state. Recent studies, including a 2021 meta-analysis, show that PPRs significantly improve performance, particularly under pressure, by enhancing focus and reducing both cognitive and somatic anxiety [51]. Future studies are encouraged to explore individual differences, contextual moderators, and underlying biological mechanisms to optimize exercise prescriptions for acute psychological benefit.

## 5. Conclusions

This study provides novel evidence that a single session of moderate-to-high-intensity RT can acutely reduce symptoms of anxiety in healthy physically active individuals. These findings extend previous literature by demonstrating that the acute psychological benefits of exercise are not restricted to low-intensity or aerobic modalities, instead, they can also be elicited through more demanding strength-based protocols, especially when performed by trained individuals in structured, familiar environments. The absence of significant improvements in the control group further emphasizes the importance of exercise intensity and neurophysiological activation in driving affective change. Taken together, these results support the use of resistance training not only as a long-term strategy for mental health promotion but also as a practical short-term tool for mood regulation in athletic populations.

## Figures and Tables

**Table 1:** Descriptive and inferential statistics (mean ± SD, median, Wilcoxon Z, *p*, and effect size r) for HADS-Anxiety (HADS-A) and HADS-Depression (HADS-D) scores in the E and C groups, measured before (Pre) and after (Post) the intervention.

Group	Status	Hospital Anxiety and Depression Scale (HADS)				
		HADS-A				
		mean ± s.d.	median	Z	*p*	r
E (*n* = 30)	Pre	6.37 ± 3.00	6	−3.3	<0.001	−0.7
	Post	4.67 ± 3.02	4			
C (*n* = 26)	Pre	5.69 ± 2.11	6	−1.5	0.139	−0.4
	Post	5.15 ± 1.91	5			
		HADS-D				
		mean ± s.d.	median	Z	*p*	r
E (*n* = 30)	Pre	4.43 ± 2.30	4.5	−2.8	<0.005	−0.6
	Post	3.37± 2.41	3.5			
C (*n* = 26)	Pre	4.58 ± 2.16	5.5	−1.5	0.135	−0.4
	Post	4.04 ± 1.84	4			

## Data Availability

The data that support the findings of this study are available from the corresponding authors, A.M. and D.M., upon reasonable request. The data are not publicly available due to privacy and ethical restrictions.
